# Shared and Distinct Time–Space Experiences Along the Psychotic-Affective Continuum

**DOI:** 10.1093/schbul/sbag027

**Published:** 2026-06-04

**Authors:** Stephan Lechner, Filipe Arantes-Gonçalves, Stefan Fritze, Jonas Daub, Geva A Brandt, Dusan Hirjak, Lilian Konicar, Georg Northoff

**Affiliations:** Brain and Mind Research Institute, The Royal’s Institute of Mental Health Research, University of Ottawa, ON K1Z 7K4, Ottawa, ON, Canada; Research Group Neuroinformatics, Faculty of Computer Science, University of Vienna, 1090, Vienna, Austria; Vienna Doctoral School Cognition, Behavior and Neuroscience, University of Vienna, 1010, Vienna, Austria; Doctoral Program in Medicine, Faculty of Medicine, University of Porto, 4200-450, Porto, Portugal; Department of Psychiatry and Psychotherapy, Central Institute of Mental Health, Medical Faculty Mannheim, University of Heidelberg, 68159, Mannheim, Germany; Department of Psychiatry and Psychotherapy, Central Institute of Mental Health, Medical Faculty Mannheim, University of Heidelberg, 68159, Mannheim, Germany; German Centre for Mental Health (DZPG), Partner Site Mannheim-Heidelberg-Ulm, 68159, Mannheim, Germany; Department of Psychiatry and Psychotherapy, Central Institute of Mental Health, Medical Faculty Mannheim, University of Heidelberg, 68159, Mannheim, Germany; German Centre for Mental Health (DZPG), Partner Site Mannheim-Heidelberg-Ulm, 68159, Mannheim, Germany; Department of Psychiatry and Psychotherapy, Central Institute of Mental Health, Medical Faculty Mannheim, University of Heidelberg, 68159, Mannheim, Germany; German Centre for Mental Health (DZPG), Partner Site Mannheim-Heidelberg-Ulm, 68159, Mannheim, Germany; ABC BRAIN LAB, Department of Child and Adolescent Psychiatry, Comprehensive Center for Pediatrics (CCP), Comprehensive Center for Clinical Neuroscience and Mental Health (C3NMH), Medical University of Vienna, 1090, Vienna, Austria; Brain and Mind Research Institute, The Royal’s Institute of Mental Health Research, University of Ottawa, ON K1Z 7K4, Ottawa, ON, Canada

**Keywords:** schizophrenia spectrum disorder, mood disorders, differential diagnosis, time and space experience, phenomenological psychopathology

## Abstract

**Background:**

Alterations in lived time and space mark many mental disorders, yet they unfold in different ways. In schizophrenia spectrum disorders (SSD), the core disturbance lies at the microstructural level: fragmented temporal synthesis and disintegration of world, self, and body. In contrast, both mood disorders (MOD) and SSD involve more accessible macro-level shifts, such as acceleration or slowing of the passage of time, fixation on the past, or blockage of the future, difficulties in social space. This overlap raises the question of how such similarities in time and space experiences of SSD and MOD can occur while, at the same time, distinguishing them.

**Study Design:**

To address this question, we used data from 26 SSD and 26 MOD patients, derived from phenomenological interviews based on the Scale for Time and Space Experience in Psychosis (STEP). We compared groups on the total STEP score and its time and space subscales, and examined differences across the 25 individual items. In a second step, we subdivided the SSD group into high and low temporospatial disturbance groups to further probe distinctions and overlaps.

**Study Results:**

The results confirm that SSD is characterized by fragmentation of temporal experience, including diminished synthesis and changes in anisotropy, fragmentation of world, self, and body. Shared items between MOD and SSD involved changes in the speed of time, shifts away from future orientation toward past and present, and spatial items related to distance and social interaction.

**Conclusions:**

Taken together, the findings indicate commonalities across disorders and disturbances that remain more characteristic of SSD.

## Introduction

Phenomenological approaches attempt to provide a thorough description of the altered structure of experience that may be distinct or overlapping across syndromes. More recently, the combination of quantitative and qualitative approaches towards patients’ experiences has seen several semi-structured interview guidelines, such as the Examination of Anomalous Self and World Experience (EASE/EAWE), and the Autism Rating Scale,^1–3^ that allow for the quantification of a plethora of experiential features that are prevalent in psychiatric syndromes. Within the field of phenomenological psychiatry, renewed interest in the subjective experience of temporality and spatiality has produced yet another semi-structured interview guide, the Scale for Time and Space Experience in Psychosis (STEP) and has further informed the creation of the Scale for Time and Space Experience in Anxiety.^4^^,^^5^ On theoretical grounds, the emphasis of these most basic experiential categories has yielded promising connections of specific structural changes in the experience of temporality and spatiality, and specific syndromes such as schizophrenia spectrum disorders (SSD), mood disorders (MOD), and anxiety disorders (AD) based on clinical reports. However, no quantitative analysis has been used yet to corroborate these claims.^6^

On the polar opposites of the SSD-MOD continuum, experiences of time are characterized by fragmentation and slowing down, respectively. As for psychotic disorders, SSD have long been described as a falling apart of the lived now, with diminished synthesis between past, present, and future on a temporal microscale akin to the phenomenological notion of retention-impression-protention,^7–9^ and an incapacity for an intentional arc into the future as in diminished anisotropy of time^10^ (see Supplementary Material for description of the STEP items used in this study). This may underlie the basic self-disturbance as well as other psychopathological dimensions in SSD^11^ and be underpinned by unstable resting state dynamics in specific frequencies.^12^ In terms of spatial experiences, SSD are often associated with alterations of the body image and, consequently, agency, a fragmentation of the world that may result in the patient feeling disoriented and overwhelmed. In contrast, mood disorder (MOD) patients, especially uni- and bipolar depression, report a general slowing-down of their inner time, a present that feels prolonged, as well as decreased capacity to project themselves into the future and being tied to the past.^8^ The slowing down of inner time has been linked to changes in the overall neuronal dynamics, which show corresponding slowing in the power spectral density as well as a decreased neuronal variability.^11^^,^^13–15^

Importantly, there exist many hybrid forms of altered time and space experience (TSE) across SSD, MOD, and AD. Psychotic symptoms can be present in around 5%-12.5% of patients with major depressive disorder (MDD),^16–18^ while depression is the most common comorbidity for patients suffering from SSD, with about a third of patients with an Axis-I SSD diagnosis receiving a secondary comorbid MDD diagnosis.^19^ Equally, changes in the structure of experience resist rigid categorization across disorders. SSD and MOD have long been discussed to form a continuous spectrum in many of their facets,^20^ with SSD and MDD representing the polar opposites, while the space between contains many hybrid diagnoses such as schizoaffective and bipolar I and II disorders,^21^ that also show substantial genetic and electrophysiological overlaps.^22–25^

Given this large overlap, we asked whether alterations of TSE show both similarities and differences in the two groups, by analyzing data collected from patients with schizophrenia spectrum disorder (psychotic group/SSD) as well as patients with MDDs bipolar and (depressed type) bipolar disorder (mood disorder group/MOD) that underwent semi-structured interviews with the STEP scale.

Previous theoretical work has previously discussed the subtle differences in time and space experience in schizophrenia and severe affective disorders, and have come to the conclusion that fragmentation or flattening is a specific structural feature of time and space experience in schizophrenia and that speed or flow of time, as well as, for example, perceived distance are abnormalities characteristic to MODs. While both can entail structural experiential changes, the fragmentary nature of these alterations in schizophrenia is further characterized by the loss of a subjective center or an altered point of view, while no such radical shifts occur in MODs. Both may experience, for example, disturbances in temporal continuity, but in a patient suffering from schizophrenia this might be due to an impaired intentional arc, that is, an incapacity to integrate retention, impression, and protention; in contrast, a discontinuity in a patient with severe depression may arise from a slowness or motivational inhibition that disconnects the patient from any experience of the future.^26^

Previous research using semi-structured interviews has demonstrated that the STEP scale encompasses discriminant and non-discriminant items when comparing SSD with MOD; however, no attempt to dissect which items are relevant for either one or both has been made.^5^ The relatively broad range of TSE probed with the STEP makes it a good potential differential tool, allowing for possible convergence across psychosis-MODs while being able to detect more uniquely psychotic experiences.

Here, we take an exploratory approach to asking which TSEs measured by the STEP are relevant specifically for schizophrenia and which are part of a broader profile that cuts across the SSD-MOD continuum. Our first specific aim is to examine whether the STEP scale is an adequate measure of both psychotic and affective dimensions. We expect the total STEP score, as well as its time and space subscales to show elevated scores in both groups, but to be more pronounced for schizophrenia patients. The *second specific aim* of this study is to identify distinct and overlapping items for SSD and MOD within the 25 STEP items. We hypothesize that especially temporal and spatial fragmentation items are defining features of SSD, whereas items related to the speed of time, as well as items associated with being blocked from the future, will not show significant group differences. We further expect more spatial items to be shared across both groups as they are more unspecific and do not exclusively relate to the psychotic experience. Our *third specific aim* is concerned with the severity of psychotic experience. The STEP scale is not only intended as a phenomenological diagnostic marker but is also aimed to serve as an index of the severity of altered time–space experience. We, therefore, further dissected the schizophrenia group into high and low temporospatial disturbance by performing a median split along the STEP total scores. We then repeated the analysis to examine distinct and overlapping items between high and low temporospatial disturbance groups and the MOD group. The goal in this analysis was to obtain a better understanding of which STEP items are more related to the psychotic as opposed to the MOD group.

## Methods

### Subjects

Semi-structured interview data were obtained from a total of 52 (26 SSD and 26 MOD, see Table S1) patients from both inpatient and outpatient clinics at the Central Institute of Mental Health (CIMH) in Mannheim, Germany, as well as two outpatient clinics associated with the Faculdade de Medicina da Universidade do Porto, Portugal. A detailed breakdown of the location-specific makeup of the sample can be found in Table S2. The German sample received diagnoses for Schizophrenia Spectrum Disorder, Major Depressive Disorder and Bipolar Disorder using the ICD-11^27^ and the German Mini Diagnostic Interview for Mental Disorders (Mini-DIPS).^28^ Diagnoses of SSD were established by experienced psychiatrists and psychologists (co-authors DH, SF, JD, and GAB) based on ICD-11 diagnostic criteria and comprehensive clinical judgment. The Mannheim cohort was recruited from the inpatient department of the CIMH. Accordingly, diagnostic decisions were informed by longitudinal clinical observations, detailed psychopathological assessments, and full access to patients’ medical records, rather than reliance on a single structured diagnostic interview. The MINI-DIPS was not used to determine the primary SSD diagnosis, as it does not capture specific SSD and has not been updated to include ICD-11 SSD criteria. Instead, the MINI-DIPS was administered exclusively to assess psychiatric comorbidities. The Portuguese sample was diagnosed in the same way but via the DSM-V^29^ instead of the ICD-11 in consensus of 2 psychiatric specialists.

The inclusion criteria for both locations also included illness duration less than 10 years, sufficient motivation and introspective skills and appropriate language to enroll in the interview., since motivation and access to rich inner life are a prerequisite for often lengthy phenomenological interviews. This was assessed via the subjective clinical impressions derived from previous clinical knowledge about the patients. The exclusion criteria for both locations included: neurological diseases; history of alcohol or substance abuse; intellectual disability; no patients under 18 or above 50 years old were included. The study was approved by the location-specific institutional review boards (Germany: The Local Ethics Committee II of the Medical Faculty Mannheim at Heidelberg University, Germany; Portugal: Ethics Committee of the Hospital de São João/Faculdade de Medicina da Universidade do Porto (Approval No.: 169-21)). Informed written consent from all study participants was obtained after all aims and procedures of the study had been explained. All STEP interviews in Portugal were conducted by FAG.

Both schizophrenia samples were included in a previous study. Here, the only addition is the inclusion of the MOD patients. While the Lisbon sample was collected chronologically prior to the Mannheim sample, the same data collection procedure was followed in Mannheim via previous discussion on procedure to minimize discrepancies between the locations. As for the remaining difference between the locations, the patients in Lisbon were recruited from outpatient clinics and included post-acute patients in stable long-term phases, whereas the Mannheim sample consisted of inpatients in more acute disease states. This is reflected in the slightly higher STEP and PANSS scores in the Mannheim sample, and especially the higher positive symptom PANSS subscale score as well as decreased discrepancy in negative symptoms, that often persist beyond acute psychosis.

### Questionnaires and Statistical Analyses

Semi-structured phenomenological interviews, like the STEP, are extensive conversations with patients that allow assessment of the idiosyncratic phenomenostructural changes in the experience of the interviewee (in this case space and time alterations), by guidelines consisting of phenomenological constructs with definitions (complemented by clinical examples) that are scored by the interviewer. Through the use of these guidelines, responses will reflect more closely the actual lived experience of the patient; something that is difficult to achieve with structured interviews that are often used in research settings for assessment of the DSM or ICD. A key difference in the phenomenological approach is that the responses to questions are worked out in cooperation with the patient, and that single answers are corroborated with additional questions through a more thorough hermeneutic process, instead of taking answers to single questions as granted. At the same time, the guidelines give clear constraints to prevent free-style interviews that could easily devolve into pure subjectivism. Semi-structured interviews have proven to be a reliable scientific tool with high interrater reliability.^30^^,^^31^

Interviews were conducted for the STEP items, resulting in scores for 25 total STEP items, including 11 time and 14 space items. Values in these responses are set between 0 (absent) and 4 (very present) for the STEP scale and were scored by expert psychiatrists based on patients’ interview responses. A list of the 25 items and their brief description can be found in the Supplementary Material. Both locations additionally collected the Positive and Negative Syndrome Scale (PANSS^32^) for all patients, regardless of diagnosis. The PANSS is a clinical 30-item scale with 3 subscales assessing positive, negative, and general symptoms. Each item is scored on a 7-point symptom severity scale ranging from 1 (absent) to 7 (severely present). For additional information detailing the location-specific makeup of the patient pool, see Table S2.

After checking for assumptions of normality and heterogeneity of variance between groups, we settled on non-parametric Mann–Whitney U tests to investigate group differences in STEP total, its subscales, and among individual STEP items, as well as PANSS total, general, positive, and negative subscales. The 25 individual STEP items were then divided into items that are distinctive for the SSD group and items that are overlapping in both groups, based on whether the respective item showed a significant difference. In this step, we used one-sided hypotheses since we expected both STEP and PANSS scores to be higher for patients with SDD, than in patients with MOD, given that they were initially designed for this patient group. Additionally, we also report effect sizes, that is, rank-biserial correlations (RBC) for group differences among the individual items. For the group differences among single items, we report uncorrected as well as conservative and liberal FRD-corrected (Benjamin–Hochberg and 2-step Benjamin–Hochberg) *P*-values but focus on uncorrected *P*-values in our interpretation since this step was exploratory.

To corroborate the group differences from the Mann–Whitney-U tests, we computed additional permutation-based ANCOVAs to examine whether the observed group differences in STEP total, time, and space were driven by data collection location, age, and sex. Permutation ANCOVAs with 5000 iterations were chosen due to their compatibility with our non-normal data and heterogeneous variances for the two groups.^33^^,^^34^

In a last step, subjects in the schizophrenia pool were subdivided at the highest value of the MOD group for the STEP total scores to create high and low temporospatial disturbance groups (hTSD vs. lTSD). We again used nonparametric alternatives (Kruskal–Wallis) to test for group differences between the 3 groups. Pairwise comparison post-hoc tests for items where Kruskal–Wallis showed significant results between the 3 groups (hTSD, lTSD, MOD) were performed using nonparametric Dunn’s tests. This analysis was performed solely on the 25 individual STEP items. Since this step was more exploratory, we report uncorrected *P*-values for this analysis. All analyses were performed in Python 3.8.

## Results

### Differences in STEP for Patients with MOD and SSD

Age and sex differences were assessed with independent *t*-tests and Chi-square test, respectively, and showed no significant difference between SSD and MOD groups. As expected, PANSS scores were higher in the SSD than in the MOD group. Non-parametric Mann–Whitney *U* tests showed differences for PANSS total, general, positive, and negative (Table S1).

**Figure 1:**
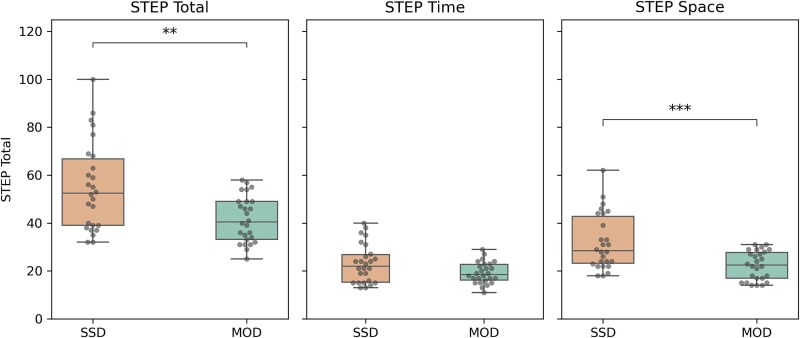
Group Differences Between Patients with MOD and SSD for STEP Total, STEP Time, and STEP Space. ^**^P < .01, ^***^P < .001.

Since our data were not normally distributed and variances were not equal between groups (see Supplementary Material), we used non-parametric alternatives, ie, Mann–Whitney U tests, to compare the 3 measures. STEP total scores (*U* = 182.0, *P*=.004, *d* = −0.46) and STEP space showed significant group differences (*U* = 168.0, *P*=.001, *r* = −0.50), while STEP time only showed a trending non-significant difference (*U* = 253.0, *P*=.06, *r* = −0.25) (Figure 1). Together, these results show that there are strong differences between SSD and MOD with respect to especially space experience.

### Difference Between Groups Persists When Controlling for Covariates

Since the sample was collected from two different locations, Portugal and Germany, we performed a permutation-based ANCOVA to control for possible confounding location and demographic effects in the group differences of STEP total, STEP space, and STEP time. *P*-values were calculated from 5000 iterations to remedy violations of the assumptions of normality and homogeneity of variance. We computed RBC as indicators of effect sizes for main group differences. For STEP total, the group effect was large, indicating higher overall STEP values in patients with SSD (*P_Perm_* = .0014, *RBC* = 0.46). Notably, the interaction between location and group was also significant (*P_Perm_* = .043), suggesting that the magnitude of the group difference varied slightly by collection site, though effect sizes cannot be computed for interactions. For STEP time, the group effect was medium, indicating a greater temporal disturbance in patients with SSD (*P_Perm_* = .035, *RBC* = 0.25). Interestingly, this differs from the unadjusted Mann–Whitney test (reported above), which only showed a trend for STEP time (*P*=.06), highlighting that adjusting for covariates revealed a significant group effect that was partially masked in the raw comparison. For STEP space, group differences were large, reflecting higher spatial disturbance in patients with SSD (*P_Perm_* = .0004, *RBC* = 0.5). Other main effects, including sex and age, and most 2-way interactions, were not significant, indicating that the observed group differences were robust and largely consistent across demographic factors and collection sites. The full results can be inspected in Table S3. Together, these results show that the covariates have no major influence on the group differences of SSD and MOD with respect to their time–space experience.

### Distinct and Overlapping STEP Items

**Figure 2:**
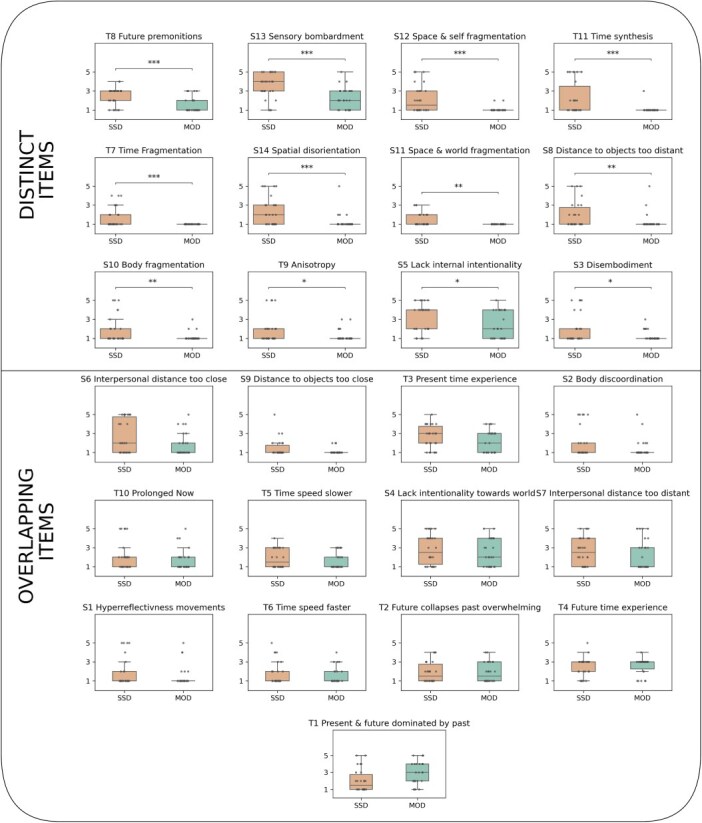
Differences Between Patients with MOD and SSD for Each Item. Items are split into two categories based on whether groups were distinct or overlapping for the item. Distinction was defined as a significant one-sided Mann–Whitney-U test. ^*^P < .05, ^**^P < .01, ^***^P < .001.

Next, we asked which of the 25 STEP items show differences between the SSD and MOD groups and which do not. Mann–Whitney U tests revealed that 13 of the 25 items showed significant differences between patients with MOD and SSD, with significantly higher scores in SSD, which are therefore specific and thus *distinct* for the SSD group (Figure 2). The remaining 12 *overlapping* items showed no significant difference between the groups. Effect sizes for distinct items were moderate (Table S4). Together, these results suggest 2 distinct types of items about time–space experience, those specific to psychosis and those shared by SSD and MOD. Broadly, items related to fragmentation, including time synthesis, anisotropy (ability for mental time travel), as well as time, space, self and body fragmentation were distinctive of the SSD group. In contrast, items related to the directness or perspective of time, such as a dominance of the future over the past and present, as well as indicators of the passage of time, like time feeling faster or slower, were shared across the psychotic-affective continuum.

### Two Different Subtypes in Schizophrenia

**Figure 3:**
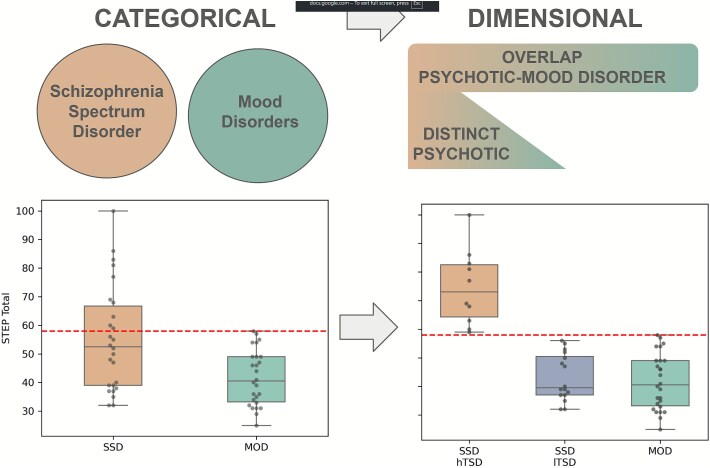
From Categorical to Dimensional Approach. Using STEP total (left), a separate schizophrenia with high temporospatial disturbance (TSD) was defined as above the threshold of the highest score of the MOD group. The remaining patients with SSD were defined as SSD with low TSD.

Next, we asked whether the SSD sample contained subgroups based on their TSEs. Therefore, we used the STEP total score and defined a threshold based on the single subject’s highest value of the MOD group. Patients with SSD who exceeded this threshold for the STEP total score were defined as SSD with hTSD, subjects below the threshold as SSD with lTSD (Figure 3).

**Figure 4:**
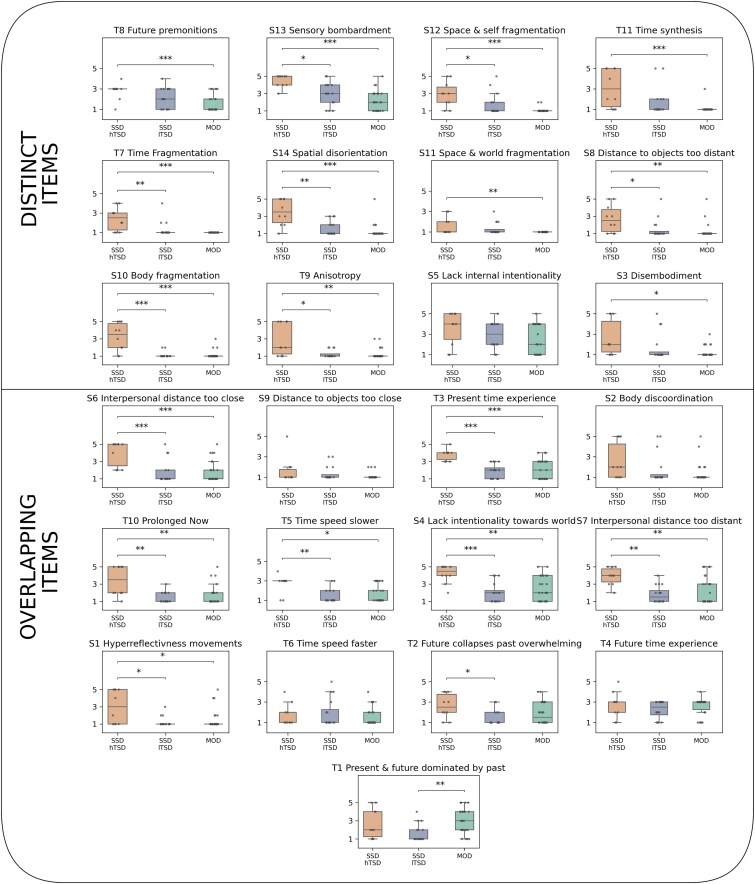
Using the Same Split into Distinct and Overlapping Items as Before, the Split into Patients with MOD and Patients with SDD with High and Low Temporospatial disturbance revealed group differences between the groups that were not present when patients with SSD were pooled. The presentation of distinct and overlapping is borrowed from Figure 2 to allow direct comparison between the two analyses. ^*^P < .05, ^**^P < .01, ^***^P < .001.

We then used these 3 groups (MOD, hTSD, lTSD) to evaluate their group differences for distinct and overlapping items. Kruskal–Wallis tests revealed significant group differences for all previously distinct items except “Future time experience,” “Time speed faster,” “Body Discoordination,” and “Distance to objects too close.” In contrast, no such group differences were shown for the overlapping items. Results for the pairwise group comparison per Dunn’s post-hoc test for each item can be found in Table S5 and Figure 4. Together, these results suggest indeed 2 distinct subtypes within the SSD group, 1 concerning psychosis with high scores as distinct from MODs, and 1 where psychosis scores are low and shared with those suffering from MODs.

Furthermore, Kruskal–Wallis tests revealed group differences between SSD hTSD, SSD lTSD, and MOD for PANSS total (*H* = 11.77, *P*=.003), positive (*H* = 17.18, *P*=.0002), and negative (*H* = 9.29, *P*=.01) but only a trend for PANSS general (*H* = 5.05, *P*=.08). Pairwise post-hoc comparisons using Dunn’s tests showed significant differences between MOD and SDD hTSD in PANSS total (*Z* = −2.69, *P*=.02), between MOD and SDD lTSD for PANSS total (*Z* = −2.88, *P*=.01), positive (*Z* = −3.94, *P*=.0001), and negative (*Z* = −2.75, *P*=.02), while no significant differences were found between hTSD and lTSD SDD groups (Table S5).

Figure 4 shows the results of the group differences per item. The figure is grouped by the same distinct and overlapping items as in Figure 2. Interestingly, splitting the SSD group into those distinct vs. overlapping with the MOD group revealed subtle differences that were not visible when patients with SSD were taken as one cohesive group.

One distinct item showed no significant group differences in this analysis of the subtypes (“Lack of internal intentionality,” which represents a loss of motivation or engagement with the environment), whereas four overlapping items did not show any group differences (“Distance to objects too close,” “Body discoordination,” “Time speed faster,” and “Future time experience”). Interestingly, some overlapping items where no difference was found for the MOD vs. SSD group showed differences between the SSD high TSD group and both MOD and SSD low TSD groups (“Interpersonal distance too close,” “Present time experience,” “Prolonged now,” “Time speed slower,” “Lack of intentionality towards world,” “Interpersonal distance too distant,” and “Hyperreflectivness of movements”). Unsurprisingly, none of the overlapping items had significant differences between patients with MOD and low TSD-SSD.

## Discussion

**Figure 5:**
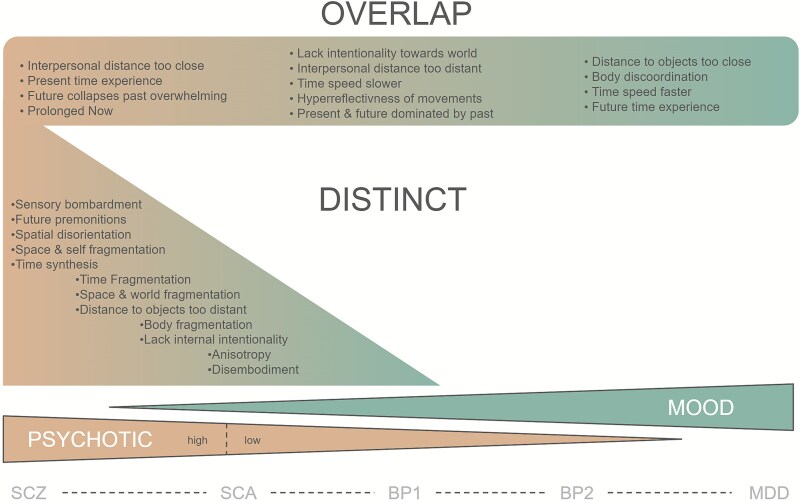
Distinct and Overlapping Features of Time and Space Experience (TSE) Across the SSD-MOD Continuum. Items are sorted according to Figure 2. Indentation of distinct items is related to RBC effect sizes.

We investigated the specificity of various phenomenological time and space disturbances assessed by the Scale for Space and Time Experience in Psychosis (STEP). In contrast to other established phenomenological interview guidelines (like the EASE and EAWE), the STEP focuses specifically on alterations of space and time experience (see Supplementary Material for a more detailed discussion). Our goal was to examine both shared and potentially differentiating items in SSD and MODs. We found a differentiating pattern for the 25 items (Figure 5). Our results show that items related to time and space fragmentation are more distinct for SDD, while changes in orientation, duration, and speed of time seem to be shared features of both SDD and MOD. When splitting the SDD into high and low temporospatial disturbance groups, distinct items appear consistently specific for the high TSD group.

TSEs have been reported for both groups, albeit with a very different focus within this experiential dimension. MODs have long been described as showing abnormal time experience,^35–37^ with these time distortions extending also to bipolar depression.^13^ Stanghellini and colleagues found that especially a dominance or hyperfocus on past experiences and slowing of the passage of time were the defining features in depression.^38^ This, combined with neuroscientific investigations on neuronal speed in depression, has led to a recent hypothesis of MDD as primarily a speed disorder.^6^ In contrast, in schizophrenia, time experience is first and foremost characterized by diminished temporal synthesis of past, present, and future and hence temporal fragmentation on a micro scale, which has been suggested to underpin the basic self-disturbance in schizophrenia.^9^^,^^11^^,^^38–40^

Our results are well in line with these descriptive and experimental accounts. In our analysis, SSD showed stronger deficits in items like “Time synthesis,” “Time fragmentation,” and “Anisotropy.” In a previous study, which reported on the SSD data used in this research, a similar grouping of items was termed time–space synthesis, which we related to core psychotic symptoms.^11^ This category also included spatial items such as “Space fragmentation,” “World fragmentation,” “Self fragmentation,” and “Body fragmentation.” Hence, time–space synthesis is manifest in SSD as the experience of fragmentation of both time and space. In this previous work, we also presented two other groupings, “Time perspective and speed” (TPS) and “Social Space” (SoSp). We tentatively related TPS items to depressive comorbidities in schizophrenia, which is in line with our presented results here: Experiences related to the speed of time (“Time speed slower” and “Time speed faster”), as well as being blocked from the future and an overemphasis on the past (“Future collapses due to past being overwhelming”, “Future time experience”, “Present & future dominated by the past”), may represent experiences that are present in both SSD and MOD. Altered experience in SoSp was previously discussed as a possible anxiety comorbidity in schizophrenia. Items in this category are present in both distinct and overlapping items. These findings can be understood as quantitative corroboration of previous theoretical work that separated the experiences along similar lines for schizophrenia and severe MODs.^26^

Last, our exploratory subdivision of the patients with SSD along the maximum value of the patients with MOD in the STEP total scale allowed us to inspect which items contributed most to the distinctly psychotic categories. When splitting patients with SSD into hTSD and lTSD subgroups, several items that appeared as overlapping in the previous analysis showed distinction for the high TSD group. Among them were items that fit well with other phenomenological descriptions. First and foremost, some items that were previously classified as overlapping are well in line with the often discussed “loss of vital contact with reality,” such as “Present time experience,” which denotes feelings of sharing a present with others, or changes in the experience of interpersonal space. In contrast, the experience of “Distance of inanimate objects being too close” or “too distant” remained a shared feature across the spectrum. Another item, which has often been discussed to be a core feature in schizophrenia and that appears as distinct when splitting hTSD from lTSD SSD patients, is “Hyperreflectivness of movements,” which probes for abnormal awareness of otherwise automatic and transparent bodily processes. Hyperreflexivity has been discussed as the phenomenological core of the schizophrenia self-disorder.^41^^,^^42^ One item that showed elevated values in hTSD SSD patients in comparison to both MOD and lTSD SSD patients resists clinical intuition: “Time speed slower”. The slowing of time is commonly discussed as a main phenomenal feature in uni- and bipolar depression.^13^^,^^38^ What emerges as the core overlapping items in this more fine-grained analysis are items related to the future. While the experienced “Collapse of the future due to the past being overwhelming” is still elevated in hTSD in comparison to the other 2 groups, the fearful experience of the future per se (“Future time experience”) remains overlapping. The “Future being dominated by the past” appears to be perhaps even closer to a distinct affective/MOD item.

Our sample was recruited from both inpatient and outpatient settings. This raises the question about the distinction between trait and state phases of both poles of the SSD-MOD continuum. Particularly, there are some items in the STEP scale which can be difficult to distinguish between patients with SSD in both prodromal and post-acute phases and patients with MOD in an acute phase. Especially item T3 (Present time experience) and S4 (Lack of intentionality towards the world), which are closely related to trait features of SSDs, may sometimes be difficult to disentangle from psychomotor retardation in acute depression.^43–45^ This may result in the partial overlap between the two poles that we have laid out in this study. Even more importantly, symptoms may be conceived of as final common pathways in different clinical syndromes. This makes a key argument to link the symptoms to different trait markers, according to different diseases. In this light, the French psychiatrist Eugene Minkowski highlighted how depressive symptoms in schizophrenia are not merely secondary manifestations, but intrinsic to the disorder, deeply rooted in time and space distortions in subjective experience.^46^

Great efforts in current psychiatry are dedicated to the identification of diagnostic markers to meaningfully categorize patients who often show complex combinations of symptoms into clearly distinguished syndromes. These efforts span a vast array of explanatory levels, such as genetic, metabolic, proteomics, psychological, and behavioral.^47–53^ While many such markers have been identified on all these levels, it is by now clear that no single marker will be sufficient for such a categorization, due to the prevalence of group overlaps and high interpersonal variability within diagnostic groups. Instead, categorization across continua may be achieved by the combination of several of these markers. If this approach of using a combination of various markers spanning various levels is to bear fruit in the future, it is key to cover all bases. Recent shifts in psychiatry have emphasized the neglect and simultaneous importance of the lived experience in psychiatric disorders.

Taking into account the lived time and space subjective experience in psychiatric disorders may help clinicians to grasp how these disturbances are manifested across different psychopathological layers (time and space deep structure, self-disturbance, and descriptive surface symptoms). In a longitudinal course of their psychopathology, patients have different trajectories which are influenced by different time and space abnormal experiences. However, one should always keep in mind that all the upper psychopathological layers are indeed different manifestations of the deepest spatiotemporal layer. That is why, in SSD, MOD, and AD symptoms frequently have some direct relationship with the lack of vital contact with reality, which is manifested in time and space alterations. The same holds true for the two SSD subtypes described in this work. We tentatively have the clinical impression that the subtype of high temporal–spatial disturbance may even be more closely related to the core Gestalt of schizophrenia.^54^

Empirical approaches toward phenomenological psychopathology seem increasingly useful for highlighting the high-dimensional overlaps between syndromes that are still often discussed as natural kinds.^55^ Especially, temporality has been suggested as a potential overarching structural change across several psychiatric disorders that may be expressed in a nuanced way across dimensions.^56^ Our findings are in agreement with the observation that SSD is characterized by time and space fragmentation, and MODs are characterized mainly by abnormal orientation towards the future and, to a lesser extent, a slowing down of time. Ever more nuanced descriptions within these broader categories of TSEs might help improve groupings for neuroscientific investigations or the establishment of tailored therapeutic approaches in precision psychiatry by finding pheno-phenotypes within previously broad categories.^57^

### Limitations

Due to the time-consuming nature of phenomenological interviews, the patient pool used for this study is rather small. This is a common problem for studies of this sort. The analyses here are exploratory but can probably inform future phenomenological differentiation of TSEs in psychiatric disorders. Interviews also require patients that can report on their inner lives in great details. This calls into question the generalizability of the results. However, we believe that the insights provided by patients with introspective capabilities extend to the larger patient populations. In any case, this is not a shortcoming specific to this study but constitutes a general limitation in any study using phenomenological interviews. Last, inter-rater reliability was not assessed in this study. The STEP is a relatively new tool, and future studies will address this shortcoming more explicitly to bolster confidence. While extensive communication between the researchers conducting and scoring the STEP was present, potential site-related differences in rating practices may still have contributed to the observed association between STEP total score and collection site.

## Conclusion

This study provides a novel empirical mapping of the phenomenological structure of time and space experience across the schizophrenia spectrum and MODs. We identify a core, psychosis-specific profile characterized by microstructural fragmentation—including diminished temporal synthesis and disintegration of world, self, and body—that is distinct from MOD. The observed diagnostic overlap is not random but is structured around a transdiagnostic profile of macro-level alterations in time speed, orientation, and social space. Grouping patients with SSD by their TSE severity revealed a further distinction: a subgroup with low disturbance that phenotypically converges with MOD, and a subgroup with high disturbance that exhibits the severe, fragmentation-based signature specific to psychosis. These findings advance beyond broad clinical description, demonstrating that quantitative approaches to phenomenological data can dissect experiential continua and validate theoretical constructs of basic experiential disturbances.

## Supplementary Material

supplement_clean_sbag027

## References

[1] Parnas J, Møller P, Kircher T, et al. EASE: examination of anomalous self-experience. *Psychopathology.* 2005;38:236-258. 10.1159/000088441 16179811

[2] Sass LA, Pienkos E, Skodlar B, et al. EAWE: examination of anomalous world experience. *Psychopathology.* 2017;50:10-54. 10.1159/000454928 28268224

[3] Stanghellini G, Ballerini M, Lysaker PH. Autism rating scale. *J Psychopathol*. 2014;20:273-285. 10.4135/9781483392271.n48

[4] Lu CJ, Goheen J, Wolman A, et al. Scale for time and space experience in anxiety (STEA): phenomenology and its clinical relevance. *J Affect Disord*. 2024;358:192-204. 10.1016/J.JAD.2024.04.099 38703910

[5] Arantes-Gonçalves F, Wolman A, Bastos-Leite AJ, Northoff G. Scale for space and time experience in psychosis: converging phenomenological and psychopathological perspectives. *Psychopathology.* 2022;55:132-142. 10.1159/000519500 34872083

[6] Northoff G . Beyond mood — depression as a speed disorder: biomarkers for abnormal slowness. *J Psychiatry Neurosci*. 2024;49:E357-E366. 10.1503/JPN.240099 39455088 PMC11530267

[7] Minkowski E . La Schizophrénie. Psychopathologie Des Schizoides et Des Schizophrénes, 1927.

[8] Stanghellini G, Ballerini M, Presenza S, et al. Psychopathology of lived time: abnormal time experience in persons with schizophrenia. *Schizophr Bull*. 2016;42:45-55. 10.1093/schbul/sbv052 25943123 PMC4681541

[9] Vogel DHV, Falter-Wagner CM, Schoofs T, Krämer K, Kupke C, Vogeley K. Flow and structure of time experience – concept, empirical validation and implications for psychopathology. *Phenomenol Cogn Sci*. 2020;19:235-258. 10.1007/S11097-018-9573-Z/TABLES/3

[10] Fuchs T . The temporal structure of intentionality and its disturbance in schizophrenia. *Psychopathology.* 2007;40:229-235. 10.1159/000101365 17396049

[11] Lechner S, Sandsten KE, Hirjak D, et al. From experience to symptoms: a Multilayer Hierarchy of psychopathological dimensions in schizophrenia. *Psychopathology*. 2025. 10.1159/000547153 40587948

[12] Lechner S, Northoff G. Temporal imprecision and phase instability in schizophrenia resting state EEG. *Asian J Psychiatr*. 2023;86:103654. 10.1016/j.ajp.2023.103654 37307700

[13] Escelsior A, Amadeo MB, Inuggi A, et al. Time perception in bipolar disorder: a systematic review. *Acta Neuropsychiatr*. 2025;37:1-17. 10.1017/NEU.2024.57 PMC13130252 39846127

[14] Northoff G, Magioncalda P, Martino M, Lee HC, Tseng YC, Lane T. Too fast or too slow? Time and neuronal variability in bipolar disorder - a combined theoretical and empirical investigation. *Schizophr Bull*. 2018;44:54-64. 10.1093/schbul/sbx050 28525601 PMC5768053

[15] Lechner S, Northoff G. Abnormal resting-state EEG phase dynamics distinguishes major depressive disorder and bipolar disorder. *J Affect Disord*. 2024;359:269-276. 10.1016/J.JAD.2024.05.095 38795776

[16] Gaudiano BA, Dalrymple KL, Zimmerman M. Prevalence and clinical characteristics of psychotic versus nonpsychotic major depression in a general psychiatric outpatient clinic. *Depress Anxiety*. 2009;26:54-64. 10.1002/DA.20470 18781658 PMC3111977

[17] Ohayon MM, Schatzberg AF. Prevalence of depressive episodes with psychotic features in the general. *Population.* 2002;159:1855-1861. 10.1176/APPI.AJP.159.11.1855 12411219

[18] Wang MQ, Wang RR, Hao Y, et al. Clinical characteristics and sociodemographic features of psychotic major depression. *Ann General Psychiatry*. 2021;20:1-8. 10.1186/S12991-021-00341-7/TABLES/4 PMC8004453 33771161

[19] Etchecopar-Etchart D, Korchia T, Loundou A, et al. Comorbid major depressive disorder in schizophrenia: a systematic review and meta-analysis. *Schizophr Bull*. 2021;47:298-308. 10.1093/SCHBUL/SBAA153 33252130 PMC8451068

[20] Marneros A. The paradigm of overlapping affective and schizophrenic spectra: schizoaffective conditions. In: Marneros A, Akiskal HS, eds. The Overlap of Affective and Schizophrenic Spectra. Cambridge University Press; 2007:1-24, 10.1017/CBO9780511544040.002.

[21] Schultze-Lutter F, Schimmelmann BG, Klosterkötter J, Ruhrmann S. Comparing the prodrome of schizophrenia-spectrum psychoses and affective disorders with and without psychotic features. *Schizophr Res*. 2012;138:218-222. 10.1016/J.SCHRES.2012.04.001 22551680

[22] Rosen C, Marvin R, Reilly JL, et al. Phenomenology of first-episode psychosis in schizophrenia, bipolar disorder, and unipolar depression: a comparative analysis. *Clin Schizophr Relat Psychoses*. 2012;6:145-151. 10.3371/CSRP.6.3.6 23006239

[23] Culbreth AJ, Foti D, Barch DM, Hajcak G, Kotov R. Electrocortical responses to emotional stimuli in psychotic disorders: comparing schizophrenia Spectrum disorders and affective psychosis. *Front Psychiatry*. 2018;9:413067. 10.3389/FPSYT.2018.00586/BIBTEX PMC6250820 30505284

[24] Witt SH, Streit F, Jungkunz M, et al. Genome-wide association study of borderline personality disorder reveals genetic overlap with bipolar disorder, major depression and schizophrenia. *Transl Psychiatry*. 2017;7:e1155. 10.1038/TP.2017.115;TECHMETA 28632202 PMC5537640

[25] Forstner AJ, Hecker J, Hofmann A, et al. Identification of shared risk loci and pathways for bipolar disorder and schizophrenia. *PLoS One*. 2017;12:e0171595. 10.1371/JOURNAL.PONE.0171595 28166306 PMC5293228

[26] Sass LA, Pienkos E. Space, time, and atmosphere a comparative phenomenology of melancholia, mania, and schizophrenia. *Part II J Conscious Stud*. 2013;20:131-152.

[27] World Helath Organization . International Statistical Classification of Diseases and Related Health Problems, 11th edn. World Health Organization, 2019.

[28] Margraf J, Cwik JC. Mini-DIPS open access: Diagnostisches Kurzinterview Bei Psychischen Störungen. *[Mini-DIPS Open Access: Diagnostic Interview for Mental Disorders]. Forschungs-Und Behandlungszentrum für Psychische Gesundheit*. Ruhr-Universität Bochum, 2017.

[29] American Psychiatric Association . Diagnostic and Statistical Manual of Mental Disorders, 5th ed Arlington, VA: American Psychiatric Association, 2013. 10.1176/appi.books.9780890425596

[30] Vollmer-Larsen A, Handest P, Parnas J. Reliability of measuring anomalous experience: the Bonn scale for the assessment of basic symptoms. *Psychopathology.* 2007;40:345-348. 10.1159/000106311 17657133

[31] Nordgaard J, Sass LA, Parnas J. The psychiatric interview: validity, structure, and subjectivity. *Eur Arch Psychiatry Clin Neurosci*. 2013;263:353-364. 10.1007/s00406-012-0366-z 23001456 PMC3668119

[32] Kay SR, Fiszbein A, Opler LA. The Positive and Negative Syndrome Scale (PANSS) for schizophrenia. *Schizophr Bull*. 1987;13:261-276. 10.1093/schbul/13.2.261 3616518

[33] Anderson MJ . A new method for non-parametric multivariate analysis of variance. *Austral Ecol*. 2001;26:32-46. 10.1111/J.1442-9993.2001.01070.PP.X

[34] Winkler AM, Ridgway GR, Webster MA, Smith SM, Nichols TE. Permutation inference for the general linear model. *Neuroimage.* 2014;92:381-397. 10.1016/J.NEUROIMAGE.2014.01.060 24530839 PMC4010955

[35] Wyrick RA, Wyrick LC. Time experience during depression. *Arch Gen Psychiatry*. 1977;34:1441-1443. 10.1001/ARCHPSYC.1977.01770240067005 263814

[36] Bschor T, Ising M, Bauer M, et al. Time experience and time judgment in major depression, mania and healthy subjects. A controlled study of 93 subjects. *Acta Psychiatr Scand*. 2004;109:222-229. 10.1046/J.0001-690X.2003.00244.X 14984395

[37] Blewett AE . Abnormal subjective time experience in depression. *Br J Psychiatry*. 1992;161:195-200. 10.1192/BJP.161.2.195 1355690

[38] Stanghellini G, Ballerini M, Presenza S, Mancini M, Northoff G, Cutting J. Abnormal time experiences in major depression: an empirical qualitative study. *Psychopathology.* 2017;50:125-140. 10.1159/000452892 27978520

[39] Stanghellini G, Fernandez AV, Ballerini M, et al. Abnormal space experiences in persons with schizophrenia: an empirical qualitative study. *Schizophr Bull*. 2020;46:530-539. 10.1093/schbul/sbz107 31784743 PMC7147594

[40] Kent L, Nelson B, Northoff G. Can disorders of subjective time inform the differential diagnosis of psychiatric disorders? A transdiagnostic taxonomy of time. *Early Interv Psychiatry*. 2023;17:231-243. 10.1111/eip.13333 36935204

[41] Sass L, Feyaerts J. Schizophrenia, the very idea: on self-disorder, hyperreflexivity, and the diagnostic concept. *Schizophr Res*. 2024;267:473-486. 10.1016/J.SCHRES.2024.03.022 38693032

[42] Feyaerts J, Nelson B, Sass L. Self-disorders in schizophrenia as disorders of transparency: an exploratory account. *Philos Psychol*. 2025;38:49-76. 10.1080/09515089.2024.2325545

[43] Hirjak D, Kubera KM, Thomann PA, Wolf RC. Motor dysfunction as an intermediate phenotype across schizophrenia and other psychotic disorders: progress and perspectives. *Schizophr Res*. 2018;200:26-34. 10.1016/J.SCHRES.2017.10.007 29074330

[44] Hirjak D, Mittal VA, Meyer-Lindenberg A, Northoff G. Future of sensorimotor and psychomotor dysfunction in mental disorders: emerging tools, methodological challenges, and the road ahead. *American Journal of Psychiatry*. 2025;182:807-812. 10.1176/APPI.AJP.20250533 40887954

[45] Hirjak D, Walther S, Mittal VA. Positive and negative falls short: why any attempt to rename schizophrenia must include careful consideration of the sensori-/psychomotor domain. *Eur Arch Psychiatry Clin Neurosci*. 2025;16:1-3. 10.1007/S00406-025-02108-7/METRICS PMC13002702 40956420

[46] Cunha F, Carreiro Borges S, Madeira L. Revisiting Eugène Minkowski’s concept of schizophrenic melancholia. *Hist Psychiatry*. 2025;36:269-278. 10.1177/0957154X251356412 40716067

[47] Kelsoe JR . The overlapping of the spectra: Overlapping genes and genetic models. In: Marneros A, Akiskal HS, eds. The Overlap of Affective and Schizophrenic Spectra. Cambridge University Press; 2007:25-42, 10.1017/CBO9780511544040.003.

[48] Frankenburg FR . The role of one-carbon metabolism in schizophrenia and depression. *Harv Rev Psychiatry*. 2007;15:146-160. 10.1080/10673220701551136 17687709

[49] Fineberg SK, Leavitt J, Deutsch-Link S, et al. Self-reference in psychosis and depression: a language marker of illness. *Psychol Med*. 2016;46:2605-2615. 10.1017/S0033291716001215 27353541 PMC7944937

[50] Torrey EF, Barci BM, Webster MJ, Bartko JJ, Meador-Woodruff JH, Knable MB. Neurochemical markers for schizophrenia, bipolar disorder, and major depression in postmortem brains. *Biol Psychiatry*. 2005;57:252-260. 10.1016/J.BIOPSYCH.2004.10.019 15691526

[51] Shin D, Rhee SJ, Shin D, et al. Integrating proteomic and clinical data to discriminate major psychiatric disorders: applications for major depressive disorder, bipolar disorder, and schizophrenia. *Clin Transl Med*. 2022;12:e929. 10.1002/CTM2.929 35758551 PMC9235346

[52] Domenici E, Willé DR, Tozzi F, et al. Plasma protein biomarkers for depression and schizophrenia by multi analyte profiling of case-control collections. *PLoS One*. 2010;5:e9166. 10.1371/JOURNAL.PONE.0009166 20161799 PMC2820097

[53] Taurines R, Dudley E, Grassl J, et al. Proteomic research in psychiatry. *J Psychopharmacol*. 2011;25:151-196. 10.1177/0269881109106931 20142298

[54] Parnas J . The core gestalt of schizophrenia. *World Psychiatry*. 2012;11:67-69. 10.1016/j.wpsyc.2012.05.002 22654930 PMC3363374

[55] Fernandez AV . Phenomenology and dimensional approaches to psychiatric research and classification. *Philos Psychiatry Psychol*. 2019;26:65-75. 10.1353/PPP.2019.0004

[56] Fuchs T, Pallagrosi M. Phenomenology of temporality and dimensional psychopathology. *Dimens Psychopathol*. 2018;30:287-300. 10.1007/978-3-319-78202-7_10

[57] Malhi GS, Parker GB, Greenwood J. Structural and functional models of depression: from sub-types to substrates. *Acta Psychiatr Scand*. 2005;111:94-105. 10.1111/J.1600-0447.2004.00475.X 15667428

