# Supplementary material for: Shared and Distinct Time–Space Experiences Along the Psychotic-Affective Continuum
Source: Schizophr Bull. 2026 Jun 4;52(3):sbag027. doi: 10.1093/schbul/sbag027 (PMC13235724; doi:10.1093/schbul/sbag027)
Supplement: supplement_clean_sbag027 [file supplement_clean_sbag027.docx]

The following is a list of brief descriptions for all 25 STEP items. For respective references and corresponding EASE, EAWE and ARS items please see the original paper on the STEP scale by Arantes-Goncalves et al. 2021

**Space Items**

**Space 1**. Hyperreflectiveness of movements: Patients have to intensively concentrate on every single movement, because, otherwise, they are unable to fulfil their actions

**Space 2**. Body discoordination: Body actions are not synchronized and constituted as a whole, but rather pieced together as successions of singular, fragmented movements with rigid, angular, and slow features.

**Space 3.** Disembodiment: Patients describe themselves as deanimated bodies (cyborgs) or disembodied spirits (scanners). They often experience themselves as robots or human machines, thus becoming passive spectators of their bodies.

**Space 4**. Lack of intentionality toward the external world: A pervasive or frequently recurrent sense of inexplicable mental or physical fatigue, dampening of immediate aliveness, diminished energy, spontaneity, lack of “Élan vital”.

**Space 5**. Lack of internal intentionality: A subjective disturbance of thought initiative, “thought energy”, or intellectual purpose. Difficulties in planning and structuring a specific task, such as cooking or writing.

**Space 6**. Interpersonal distance too close: Immediate feeling of being invaded, overrun, flooded, constrained, or threatened by others. To be, somehow, in a passive, dangerously exposed position at the mercy of others. There might be emotional paroxysms. Even familiar people might be experienced as unfamiliar, strange, and threatening in touch

**Space 7**. Interpersonal distance too distant: Feelings of being detached from others, such as feeling a sense of remoteness, rejection or even of being ignored by others. Patients get too closed in their inner world.

**Space 8**. Distance to physical objects and space is too distant: External space and physical objects are perceived as too distant. Space might also be perceived as infinite, unreal, and threatening.

**Space 9.** Distance to physical objects and space is too close: Space and objects are felt as too close. Sometimes, the patient is unable to determine where the body ends and the outside world begins. A physical intermingling, in which external objects seem to be inside or merged within the patient’s body. In some cases, the patient might feel invaded by the outside world.

**Space 10.** Body fragmentation: It may include the perception of constriction of single body parts or the whole body; alternatively, those parts (or the whole body) may be perceived as becoming thinner, shorter, enlarged, pressed down, or diminished. Also, body parts may be experienced as strange, alien, lifeless, isolated, separated from each other, dislocated, or even as non-existent. These can be accompanied by feelings of perplexity and meaninglessness.

**Space 11**. Space and world fragmentation: A scene, landscape, or the surrounding world loses its cohesive unity; objects seem to be isolated, disconnected from their context, and less meaningful. These are no longer related to one another or become literally separated. Patients may try to compensate for the aforementioned abnormalities by searching or putting everything together, irrespective of the corresponding nonsense.

**Space 12**. Space and self-fragmentation: Emotions are projected into parts of the body. Emotional experiences may be felt as artificial. Because of these projections, the outside space may be experienced as disconnected from the patients.

**Space 13**. Sensory bombardment: Patients feel the external space as too stimulating and overwhelming. They feel confused and disoriented because they seem to experience sensory bombardment. In other words, patients experience too much stimuli from everywhere at the same time.

**Space 14**. Spatial disorientation: In a familiar location, subjects feel completely lost and disoriented. Spatial perspectives become misleading. These experiences are accompanied by subjective states of disorientation and confusion.

**Time Items**

**Time 1**. Present and future are dominated by the past: Present and future are dominated by the past, mainly because of the feeling of guilt requiring punishment

**Time 2**. Future directedness collapses, because the past is overwhelming: There is a dysconnection between past and present/ future, due to excess of traumatic memories or perplexity, meaninglessness, or even the feeling of the past as nonexistent

**Time 3**. Present time experience: Present is experienced with difficulties of sharing a normal life with others, or with significant passivity.

**Time 4**. Future time experience: The future is experienced as being very difficult, lacking any significance, or even felt as nonexistent, possibly because of worries and fears

**Time 5**. Time speed is slower: Time speed is slower than that perceived by others; slower in general (along with feelings of perplexity and meaninglessness), or even completely stopped (EAWE 2.2.1). Here, the key feature is the loss of the dynamics of time

**Time 6**. Time speed is faster: Time speed is experienced as faster across different levels (e.g., faster than that perceived by other people, faster in general, along with perplexity and meaninglessness)

**Time 7**. Time fragmentation: Time is experienced as fragmented, and with dysconnectivity in its synthesis. This may manifest as time being felt as a series of photographs, along with feelings of astonishment and perplexity or even without any associated significance

**Time 8**. Future premonitions: Future is experienced as a series of premonitions: sometimes something threatening is perceived as being about to happen, and other times the future is felt as indifferent or even as nonexistent at all

**Time 9**. Anisotropy: Mental time travelling (across past, present, and future) is difficult for patients, who have the feeling that they are stuck in the present and cannot synchronize with their environment

**Time 10**. The present is felt as too prolonged (“longer now”): Events in time, which are perceived by other people as distinct, are often felt by patients as similar and simultaneous

**Time 11**. Time synthesis: Time synthesis is lost, because it seems that time is concentrated in the present moment

**Relationship of space and self in “Space and self fragmentation”**

A disturbance in spatial experience in schizophrenia often follows a progression from the self to the world. The initial disruption is a fragmentation of the self at the emotional and experiential level. This fragmented state of self is frequently accompanied by a fragmentation of inner space, meaning the patient's sense of their own body as a coherent vessel breaks down. These two fragmentations—of self and of inner bodily space—are typically observed together and are difficult to separate. In more severe cases, this disintegration may extend into external space. The patient perceives the external world as fractured or fragmented. Clinically, the fragmentation of the self appears first, the fragmentation of inner space follows from it, and the fragmentation of the external world is a subsequent, more profound disturbance.

**Relationship of “Time fragmentation” and “Time synthesis**

Time fragmentation" addresses the interruption of the normal flow of time consciousness as retention - primal impression - portention. Here, a clear progression in time is still possible, but the intuitive flow between moments becomes impaired, leading to experiences like perceiving separate moments as snapshots that, to some extent, are decoupled from their temporal context. “Time synthesis” addresses the consequence of this temporal fragmentation, when the flow of time consciousness has seized altogether (at least temporarily), and past and future do not exist anymore and hence only the present remains. This item is also intimately related to the time item 10 the “prolonged now. (Mancini et al., 2015).

**Phenomenological Overlap Between STEP, EASE, and EAWE** -
Alterations in time and space experience are at least as old as modern psychopathology itself. Examples can be found in Bleuler, Kraepelin, Blankenburg, and Konrad, among others. Understandably, some of these themes have been introduced into other (and similar) semi-structured interview guidelines such as the EASE and the EAWE. For example, the EAWE contains domains labelled “Space and Objects” and “Time and Events”. The former contains items that relate directly to items included in the STEP. For example, items in “1.4 Visual fragmentation”, are essentially the same experiences that some of the STEP items aim for. The latter also contains items that are present in the STEP. For example, “2.1. Time or Movements Appear to Change Speed” are shared between the two scales. These are common experiences that are also present in the EASE, for example, “1.14 Disturbance in Experience of Time” probes for the same basic experiential change in the speed or flow of time. Yet, the three questionnaires do not share the same overall focus. Both the EASE and EAWE include some items related to time and space experience, while the STEP is specifically designed to exclusively quantify these experiences, with a focused emphasis on measuring alterations in the structure of time and space experience. This phenomenostructural emphasis clearly excludes content-based items that directly address, for example, visual or auditory hallucinations, that are to some extent included in EASE and EAWE. When a patient reports hallucinations, the interviewer is still interested in how they present themselves in a dynamic spatiotemporal manner. For example, a patient may describe hearing voices, but the interviewer might be interested in how these voices are perceived temporally—whether the voices occur in a continuous flow or if they are fragmented, with pauses or interruptions in the experience. They may also inquire whether the voices seem to change in temporal consistency, such as varying in volume or speed, or if they seem to emerge and disappear unpredictably, disrupting the continuity of the experience. These temporal dynamics of the hallucination, rather than just the content of the voices themselves, are central to understanding the alteration of time perception in the STEP, but are not directly addressed in the EASE or EAWE. With 25 items, the STEP is also considerably shorter and represents a distilled form for a particular subset of experiences. Since semi-structured interviews are, by definition, open, this does, however, not always translate into less time-intensive interview sessions.

Last, we also want to point to an empirically relationship between STEP and EASE, as documented in previous work, that correlated EASE and STEP and found a strong correlation between the two (r = 0.84) (Lechner et al., 2025).”

| **Supplementary Table 1. Demographic description of the sample** | | | | | | | | | | | | | | | | | |  |  |  |
| --- | --- | --- | --- | --- | --- | --- | --- | --- | --- | --- | --- | --- | --- | --- | --- | --- | --- | --- | --- | --- |
|  | **MOD** | | | | **SSD** | | | | | | **Differences** | | | | | | |  |  |  |
| *N* | 26 | | | | 26 | | | | | |  | | | | | | |  |  |  |
| Age | 38.46 ± 11.88 | | | | 35.77 ± 10.76 | | | | | | t = 0.84, p = 0.405 | | | | | | |  |  |  |
| Sex (f/m) | 16 (61.5%) 10 (38.5%) | | | | 14 (53.8%) 12 (46.2%) | | | | | | χ2 = 2.48, p = 0.116 | | | | | | |  |  |  |
| PANSS Total | 46.42 ± 13.84 | | | | 60.54 ± 16.09 | | | | | | U = 151, p = 0.001 | | | | | | |  |  |  |
| PANSS General | 27.00 ± 7.76 | | | | 31.31 ± 8.16 | | | | | | U = 227, p = 0.04 | | | | | | |  |  |  |
| PANSS Positive | 7.96 ± 1.61 | | | | 13.04 ± 5.76 | | | | | | U = 134.5, p < 0.001 | | | | | | |  |  |  |
| PANSS Negative | 11.46 ± 5.41 | | | | 16.19 ± 5.97 | | | | | | U = 173, p = 0.002 | | | | | | |  |  |  |
|  |  | | | |  | | | | | |  | | | | | | |  |  |  |
| \| **Supplementary Table 2. Location specific demographic information** \| \| \| \| \| \| --- \| --- \| --- \| --- \| --- \| \|  \| **Germany** \| \| **Portugal** \| \| \|  \| **MOD** \| **SSD** \| **MOD** \| **SSD** \| \| N \| 16 \| 17 \| 10 \| 9 \| \| Age \| 42.00 ± 13.39 \| 37.24 ± 11.71 \| 32.80 ± 5.31 \| 33.00 ± 7.99 \| \| Sex (f/m) \| 12 (75.0%) 4 (25.0%) \| 12 (70.6%) 5 (29.4%) \| 6 (60.0%) 4 (40.0%) \| 7 (77.8%) 2 (22.2%) \| \| Major Depressive  Disorder \| 11 \| - \| 9 \| - \| \| Bipolar Disorder \| 4* \| - \| 1^†^ \| - \| \| Dysthemia \| 1 \| - \| - \|  \| \| Schizophrenia \| - \| 12 \| - \| 4 \| \| Schizoaffective disorder \| - \| 4 \| - \| 1 \| \| Schizotypical Personality Disorder \| - \| 1 \| - \| 4 \| \| PANSS Total \| 53.81 ± 12.82 \| 65.18 ± 17.05 \| 34.60 ± 2.46 \| 51.78 ± 9.74 \| \| PANSS General \| 31.00 ± 7.23 \| 32.71 ± 8.51 \| 20.60 ± 2.46 \| 28.67 ± 7.14 \| \| PANSS Positive \| 8.62 ± 1.75 \| 15.94 ± 5.06 \| 6.90 ± 0.32 \| 7.56 ± 0.88 \| \| PANSS Negative \| 14.19 ± 5.31 \| 16.53 ± 6.42 \| 7.10 ± 0.32 \| 15.56 ± 5.29 \|   * All 4 bipolar patients were of depressed type (2 patients with ICD diagnosis F31.3 and 2 patients with ICD-diagnosis 31.4; ^†^ One bipolar I patient undifferentiated-type  **Description of normality and heterogeneity of variance**  The Shapiro-Wilk test revealed that data in STEP space was not normally distributed in both the MOD group (*W*(1,50) = 0.9, *p* = 0.02) and the SDD group (*W*(1,50) = 0.91, *p*= 0.02) and Levene’s test showed that variances were not equal in the two groups for STEP total (*W*(1,50) = 7.12, *p* = 0.01), STEP time (*W* = 6.97, *p* = 0.011) and STEP space (*W*(1,50) = 5.53, *p* = 0.023).  **Supplementary Table 3. Permutation-based ANOVA for STEP total, space and time** | | | | | | | | | | | | | | | | | | |  |  |
| **STEP total** | | **Sum Sq** | | **df** | | ***F*** | | | ***p*** | | | ***p (perm)*** | | | ***RBC*** | | | |  |  |
| Location | | 61.75 | | 1 | | 0.31 | | | 0.58 | | | 0.57 | | | −0.19 | | | |  |  |
| Sex | | 161.96 | | 1 | | 0.82 | | | 0.37 | | | 0.37 | | | 0.19 | | | |  |  |
| group | | 2341.75 | | 1 | | 1.91 | | | 0.00 | | | 0.0014 | | | 0.46 | | | |  |  |
| Location:Sex | | 1.57 | | 1 | | 0.01 | | | 0.93 | | | 0.93 | | |  | | | |  |  |
| group:Sex | | 33.10 | | 1 | | 0.17 | | | 0.68 | | | 0.68 | | |  | | | |  |  |
| Location:group | | 870.30 | | 1 | | 4.43 | | | 0.04 | | | 0.043 | | |  | | | |  |  |
| Age | | 239.86 | | 1 | | 1.22 | | | 0.28 | | | 0.27 | | |  | | | |  |  |
| Location:Age | | 435.16 | | 1 | | 2.21 | | | 0.14 | | | 0.15 | | |  | | | |  |  |
| Residual | | 8452.11 | | 43 | |  | | |  | | | 0.0002 | | |  | | | |  |  |
| **STEP time** | | **Sum Sq** | | **df** | | ***F*** | | | ***p*** | | | ***p (perm)*** | | | ***RBC*** | | | |  |  |
| Location | | 0.01 | | 1 | | 0.00 | | | 0.99 | | | 0.99 | | | −0.08 | | | |  |  |
| Sex | | 46.09 | | 1 | | 1.21 | | | 0.28 | | | 0.29 | | | 0.24 | | | |  |  |
| group | | 179.01 | | 1 | | 4.70 | | | 0.04 | | | 0.035 | | | 0.25 | | | |  |  |
| Location:Sex | | 11.26 | | 1 | | 0.30 | | | 0.59 | | | 0.59 | | |  | | | |  |  |
| group:Sex | | 5.58 | | 1 | | 0.15 | | | 0.70 | | | 0.70 | | |  | | | |  |  |
| Location:group | | 150.65 | | 1 | | 3.96 | | | 0.05 | | | 0.054 | | |  | | | |  |  |
| Age | | 62.56 | | 1 | | 1.64 | | | 0.21 | | | 0.21 | | |  | | | |  |  |
| Location:Age | | 58.71 | | 1 | | 1.54 | | | 0.22 | | | 0.22 | | |  | | | |  |  |
| Residual | | 1637.56 | | 43 | |  | | |  | | | 0.0002 | | |  | | | |  |  |
| **STEP space** | | **Sum Sq** | | **df** | | ***F*** | | | ***p*** | | | ***p (perm)*** | | | ***RBC*** | | | |  |  |
| Location | | 59.98 | | 1 | | 0.75 | | | 0.39 | | | 0.40 | | | −0.22 | | | |  |  |
| Sex | | 35.25 | | 1 | | 0.44 | | | 0.51 | | | 0.52 | | | 0.14 | | | |  |  |
| group | | 1225.85 | | 1 | | 5.25 | | | 0.00 | | | 0.0004 | | | 0.50 | | | |  |  |
| Location:Sex | | 21.23 | | 1 | | 0.26 | | | 0.61 | | | 0.61 | | |  | | | |  |  |
| group:Sex | | 11.49 | | 1 | | 0.14 | | | 0.71 | | | 0.71 | | |  | | | |  |  |
| Location:group | | 296.76 | | 1 | | 3.69 | | | 0.06 | | | 0.06 | | |  | | | |  |  |
| Age | | 57.42 | | 1 | | 0.71 | | | 0.40 | | | 0.41 | | |  | | | |  |  |
| Location:Age | | 174.19 | | 1 | | 2.17 | | | 0.15 | | | 0.15 | | |  | | | |  |  |
| Residual | | 3457.6 | | 43 | |  | | |  | | | 0.0002 | | |  | | | |  |  |
| **Supplementary Table 4. Group comparisons between MOD and SSD for each space and time item** | | | | | | | | | | | | | | | | | | | | |
| **Item** | | | ***W*** | | | | ***p*** |  | | ***p_corr (bh)_*** | | |  | ***p_corr (tsbh)_*** | |  | ***RBC*** | | |  |
| **DISTINCT ITEMS** | | | | | | | | | | | | | | | | | | | | |
| S13 Sensory bombardment | | | 148.5 | | | | 0.0002 | *** | | 0.001 | | | ** | 0.0002 | | *** | -0.28 | | |  |
| T8 Future premonitions | | | 158 | | | | 0.0002 | *** | | 0.001 | | | ** | 0.0002 | | *** | -0.27 | | |  |
| S14 Spatial disorientation | | | 185.5 | | | | 0.0006 | *** | | 0.003 | | | ** | 0.0003 | | *** | -0.23 | | |  |
| S12 Space & self fragmentation | | | 186 | | | | 0.0003 | *** | | 0.001 | | | ** | 0.0002 | | *** | -0.22 | | |  |
| T11 Time synthesis | | | 194 | | | | 0.0003 | *** | | 0.001 | | | ** | 0.0002 | | *** | -0.21 | | |  |
| T7 Time Fragmentation | | | 208 | | | | 0.0003 | *** | | 0.001 | | | ** | 0.0002 | | *** | -0.19 | | |  |
| S11 Space & world fragmentation | | | 234 | | | | 0.0012 | ** | | 0.004 | | | ** | 0.0005 | | *** | -0.15 | | |  |
| S8 Distance to objects too distant | | | 234.5 | | | | 0.0078 | ** | | 0.02 | | | * | 0.003 | | ** | -0.15 | | |  |
| S10 Body fragmentation | | | 240.5 | | | | 0.0097 | ** | | 0.03 | | | * | 0.003 | | ** | -0.14 | | |  |
| S5 Lack internal intentionality | | | 244.5 | | | | 0.037 | * | | 0.08 | | |  | 0.01 | | ** | -0.14 | | |  |
| T9 Anisotropy | | | 257 | | | | 0.035 | * | | 0.08 | | |  | 0.01 | | ** | -0.12 | | |  |
| S3 Disembodiment | | | 257.5 | | | | 0.039 | * | | 0.08 | | |  | 0.01 | | ** | -0.12 | | |  |
| **OVERLAPPING ITEMS** | | | | | | | | | | | | | | | | | | | | |
| S6 Interpersonal distance too close | | | 257 | | | | 0.056 |  | | 0.1 | | |  | 0.01 | | * | -0.12 | | |  |
| T3 Present time experience | | | 259.5 | | | | 0.07 |  | | 0.11 | | |  | 0.01 | | * | -0.12 | | |  |
| S2 Body discoordination | | | 276 | | | | 0.08 |  | | 0.13 | | |  | 0.02 | | * | -0.09 | | |  |
| T10 Prolonged Now | | | 277 | | | | 0.11 |  | | 0.17 | | |  | 0.02 | | * | -0.09 | | |  |
| S9 Distance to objects too close | | | 281.5 | | | | 0.07 |  | | 0.11 | | |  | 0.01 | | * | -0.08 | | |  |
| S4 Lack intentionality towards world | | | 282 | | | | 0.15 |  | | 0.18 | | |  | 0.02 | | * | -0.08 | | |  |
| S7 Interpersonal distance too distant | | | 283 | | | | 0.15 |  | | 0.18 | | |  | 0.02 | | * | -0.08 | | |  |
| T5 Time speed slower | | | 285 | | | | 0.14 |  | | 0.18 | | |  | 0.02 | | * | -0.08 | | |  |
| S1 Hyperreflectivness movements | | | 292.5 | | | | 0.15 |  | | 0.18 | | |  | 0.02 | | * | -0.07 | | |  |
| T6 Time speed faster | | | 302.5 | | | | 0.22 |  | | 0.26 | | |  | 0.03 | | * | -0.05 | | |  |
| T2 Future collapses past overwhelming | | | 339.5 | | | | 0.52 |  | | 0.56 | | |  | 0.07 | |  | 0.002 | | |  |
| T4 Future time experience | | | 381.5 | | | | 0.82 |  | | 0.85 | | |  | 0.1 | |  | 0.06 | | |  |
| T1 Present & future dominated by past | | | 475.5 | | | | 0.99 |  | | 0.99 | | |  | 0.12 | |  | 0.2 | | |  |

| **Supplementary Table 5. Results of Kruskal-Wallis Test and groupwise comparison Dunn's post-hoc tests for all items** | | | | |  |
| --- | --- | --- | --- | --- | --- |
| **Item** | **Kruskal-Wallis** | **MOD - hTSD** | **MOD - lTSD** | **hTSD - lTSD** |  |
| T1 Present & future dominated by past | H = 10.31, p = 0.0058** | Z = 0.65, p =  1.0 | Z = 3.09, p = 0.0043** | Z = 1.83, p = 0.1762 |  |
| T2 Future collapses past overwhelming | H = 7.22, p =  0.027* | Z = −1.64, p = 0.2304 | Z = 1.24, p = 0.5435 | Z = 2.49, p = 0.0216* |  |
| T3 Present time experience | H = 17.8, p = 0.0001*** | Z = -3.59, p = 0.0005*** | Z = 0.59, p = 1.0 | Z = 3.79, p = 0.0002*** |  |
| T4 Future time experience | H = 1.9, p = 0.3864 | - | - | - |  |
| T5 Time speed slower | H = 10.85, p = 0.0044** | Z = −2.58, p = 0.0119* | Z = 0.51, p = 1.0 | Z = 2.78, p = 0.0056** |  |
| T6 Time speed faster | H = 0.59, p = 0.7429 | - | - | - |  |
| T7 Time Fragmentation | H = 22.68, p =  0.0*** | Z = −3.27, p =  0.0*** | Z = -0.98, p = 0.4618 | Z = 2.25, p = 0.0032** |  |
| T8 Future premonitions | H = 14.88, p = 0.0006*** | Z = −3.39, p = 0.0008*** | Z = -2.19, p = 0.0549 | Z = 1.4, p = 0.3916 |  |
| T9 Anisotropy | H = 11.22, p = 0.0037** | Z = −2.63, p = 0.0037** | Z = -0.18, p = 1.0 | Z = 2.28, p = 0.0149* |  |
| T10 Prolonged Now | H = 11.71, p = 0.0029** | Z = −2.79, p = 0.0071** | Z = 0.46, p = 1.0 | Z = 2.94, p = 0.0041** |  |
| T11 Time synthesis | H = 17.68, p = 0.0001*** | Z = −3.16, p = 0.0001*** | Z = -1.42, p = 0.1825 | Z = 1.8, p = 0.0534 |  |
| S1Hyperreflectivness movements | H = 8.19, p = 0.0166* | Z = −2.04, p = 0.0319* | Z = 0.31, p =  1.0 | Z = 2.13, p = 0.0231* |  |
| S2 Body discoordination | H = 5.19, p =  0.0748 | - | - | - |  |
| S3 Disembodiment | H = 8.42, p = 0.0148* | Z = −2.37, p = 0.013* | Z = -0.36, p =  1.0 | Z = 1.9, p = 0.0653 |  |
| S4 Lack intentionality towards world | H = 14.02, p = 0.0009*** | Z = −3.09, p = 0.0044** | Z = 0.81, p =  1.0 | Z = 3.48, p = 0.001*** |  |
| S5 Lack internal intentionality | H = 4.54, p =  0.1031 | - | - | - |  |
| S6 Interpersonal distance too close | H = 16.58, p = 0.0003*** | Z = −3.43, p = 0.0007*** | Z = 0.4, p =  1.0 | Z = 3.48, p = 0.0005*** |  |
| S7 Interpersonal distance too distant | H = 12.55, p = 0.0019** | Z = −2.9, p = 0.0071** | Z = 0.7, p =  1.0 | Z = 3.23, p = 0.0022** |  |
| S8 Distance to objects too distant | H = 12.56, p = 0.0019** | Z = −2.75, p = 0.0012** | Z = -0.67, p =  1.0 | Z = 2.01, p = 0.0295* |  |
| S9 Distance to objects too close | H = 2.36, p = 0.307 | - | - | - |  |
| S10 Body fragmentation | H = 22.9, p =  0.0*** | Z = −3.44, p =  0.0*** | Z = -0.01, p =  1.0 | Z = 3.17, p = 0.0001*** |  |
| S11 Space & world fragmentation | H = 10.43, p = 0.0054** | Z = −1.88, p = 0.0082** | Z = -1.32, p = 0.1043 | Z = 0.69, p = 0.8103 |  |
| S12 Space & self fragmentation | H = 19.69, p = 0.0001*** | Z = −3.54, p =  0.0*** | Z = -1.36, p = 0.2679 | Z = 2.2, p = 0.0179* |  |
| S13 Sensory bombardment | H = 19.26, p = 0.0001*** | Z = −4.27, p =  0.0*** | Z = -1.79, p = 0.2006 | Z = 2.53, p = 0.0291* |  |
| S14 Spatial disorientation | H = 20.76, p =  0.0*** | Z = −3.91, p =  0.0*** | Z = -1.09, p = 0.6088 | Z = 2.75, p = 0.0042** |  |
| PANSS total | H = 11.77, p = 0.0028** | Z = −2.69, p = 0.0211* | Z = −2.88, p = 0.0118* | Z = 0.21, p =  1.0 |  |
| PANSS general | H = 5.05, p =  0.0801 | - | - | - |  |
| PANSS positive | H = 17.18, p = 0.0002*** | Z = −1.84, p = 0.1659 | Z = −3.94, p = 0.0001*** | Z = −1.4, p = 0.4314 |  |
| PANSS negative | H = 9.28, p = 0.0096** | Z = −2.1, p =  0.1036 | Z = -2.75, p = 0.0172* | Z = −0.23, p =  1.0 | |

**References**
Mancini, M., Presenza, S., Di Bernardo, L., Lardo, P. P., Totaro, S., Trisolini, F., ... & Stanghellini, G. (2014). The life-world of persons with schizophrenia. A panoramic view. Journal of Psychopathology, 20, 423-434.
